# Multimodal Integration of Spatial Information: The Influence of Object-Related Factors and Self-Reported Strategies

**DOI:** 10.3389/fpsyg.2016.01443

**Published:** 2016-09-21

**Authors:** Harun Karimpur, Kai Hamburger

**Affiliations:** Experimental Psychology and Cognitive Science, Justus Liebig UniversityGiessen, Germany

**Keywords:** multimodality, modality, wayfinding, landmark, congruency, cognitive style

## Abstract

Spatial representations are a result of multisensory information integration. More recent findings suggest that the multisensory information processing of a scene can be facilitated when paired with a semantically congruent auditory signal. This congruency effect was taken as evidence that audio-visual integration occurs for complex scenes. As navigation in our environment consists of a seamless integration of complex sceneries, a fundamental question arises: how is human landmark-based wayfinding affected by multimodality? In order to address this question, two experiments were conducted in a virtual environment. The first experiment compared wayfinding and landmark recognition performance in unimodal visual and acoustic landmarks. The second experiment focused on the congruency of multimodal landmark combinations and additionally assessed subject’s self-reported strategies (i.e., whether they focused on direction sequences or landmarks). We demonstrate (1) the equality of acoustic and visual landmarks and (2) the congruency effect for the recognition of landmarks. Additionally, the results point out that self-reported strategies play a role and are an under-investigated topic in human landmark-based wayfinding.

## Introduction

Finding our way in everyday life does not seem to be a great challenge at first glance. But, we all know how difficult it can be when we get lost (Dudchenko, 2010). Folk wisdom says: we relied on our senses; did we? How finding the right path is affected by both multisensory cues and multisensory integration (i.e., binding cues from at least two different sensory modalities to form a single, meaningful object or entity) remains an outstanding issue.

In order to address this issue, we will first exemplary describe situations in wayfinding which emphasize *the importance of modalities other than vision*. Second, we will take a closer look to reference points during wayfinding, so-called *landmarks*, in which we will describe two important aspects of these landmarks: their inherent features and the sensory channels they trigger. We will conclude that especially the latter aspect has not been the primary object of research in human wayfinding. Which is even more the case for the last topic we present: *multimodality*. Finally, in a series of two experiments we will show that there is more to wayfinding than visual cues. By examining the role of different sensory modalities and cognitive styles, we will try to gain insight into processes involved in human landmark-based wayfinding.

### The Importance of Modalities Other than Vision

In the field of spatial cognition, wayfinding is a well-investigated topic (Montello et al., 2004; Montello, 2005). However, the vast majority of research was based on the assumption that human wayfinding primarily builds on one modality, namely vision. But what about other sensory modalities? Our visual reference points (e.g., church) are often accompanied by acoustic signals (e.g., church bells). This does not only hold in real life but also in imagined situations. For example, in guided imagery, which is often used in clinical settings, participants are asked to imagine being in a certain place and describe what they see, hear and feel. Furthermore, in situations where visual cues cannot be used (e.g., visual impairment), we see that other sensory modalities can play a role in wayfinding. This is especially the case in situations in which visual input is rarely available or may not be useful. In such situations, the visual part of working memory (the visuo-spatial sketchpad; Baddeley and Hitch, 1974; Baddeley, 2003) is already occupied with other information that requires further processing. In an experimental setting, this was tested by letting participants conduct a wayfinding task as well as a secondary task (verbal, spatial, visual). For example, in the spatial secondary task condition, participants had to indicate from which direction a sound was coming (Meilinger et al., 2008). The authors could show that additional load on the visuo-spatial sketchpad interfered with wayfinding performance. The following shall describe another situation where visual cues can hardly be used.

Let us assume we learned that at the end of a hallway an illuminated exit sign is hanging from the ceiling. All of a sudden, a fire breaks out in an office and because of the smoke we can barely see anything. We hear the jarring sound of the alarm coming from loudspeakers and look out for our exit sign in order to find our way out of the hallway. The question now is: how does the combination of visual and acoustic signals influence our wayfinding performance? The findings presented in this study will be of particular interest. They not only bring us a step forward in understanding multimodality in human wayfinding but can also be applied in our everyday lives. In the aforementioned situation, intentionally placed acoustic landmarks could guide people through darkness and thick smoke (e.g., Walker and Lindsay, 2006). Or we could enhance the effectiveness of navigation systems by understanding the importance of modalities in wayfinding.

Until now we focused on the information made available in a certain situation. But, the question remains whether each and every person is able to make use of the given information? The answer is no. For example, it is obvious that blind people lack the ability to make use of any type of visual cues. For them it is well-investigated that they need to receive some type of acoustic or haptic signals in order to find their ways (e.g., Loomis et al., 1998; Loomis et al., 2002).

But, what about people without any perceptual and/or cognitive deficits? Will we find differences within such a group? The answer is yes. A major issue in this context is that performance in any cognitive task is not only determined by our perceptual and cognitive abilities, but also by so-called cognitive styles, i.e., how people construct themselves and their environment (e.g., Klein, 1951). This means that it is in psychological research also of great importance to not only focus on performance from a third-person view, but also to take individual strategies and preferences into account (e.g., Wedell et al., 2014). These strategies and preferences affect the cognitive processes taking place and the resources that need to be applied in spatial and other tasks (Pazzaglia and Moè, 2013). For our purposes here the distinction between ‘object-learners’ and ‘route-learners’ will be of major importance. As the terms indicate object-learners are those people who preferably focus on landmark objects, while the route-learners should concentrate more on the path, encoding spatial information as mere directional instructions: right, left, left, straight, right, and so on. Route encoders should perform worse on recognition tasks due to the fact that they normally try to neglect the given landmark-objects, since they perceive them as distracting (Hamburger and Röser, 2014). However, it is well-known that landmarks represent valuable spatial information.

### Landmarks

How do we reach our destination? According to Montello (2005) the key components of navigation are locomotion and wayfinding. In this study, we will focus on wayfinding and particularly on one important aspect, that is how landmarks help us find our way (landmark-based wayfinding). Landmarks can typically be described as features or reference points in space that stand out from the environment (Lynch, 1960; Caduff and Timpf, 2008). At decision points (e.g., intersections), they serve as cues helping us to make the right choice and as a consequence to take the correct direction. Additionally, between decision points, they can serve as so-called maintenance landmarks which ensure individuals that they are still on the correct path (Presson and Montello, 1988).

The topic of landmark-based wayfinding is nowadays often investigated by using virtual environments. At first glance they seem to be much different from real world sceneries. They often seem to be too simple or too complex. And the lack of visual-vestibular feedback is also a valid point of criticism. These are reasons why experiments conducted in such environments may lack ecological validity. However, an advantage of such environments is that, depending on the investigated problem, the degree of complexity of a scene can be adjusted. It allows researchers to manipulate particular aspects and thus leads to a high degree of experimental control. The role of virtual environments remains subject to debate which is why we addressed this topic also in the discussion section.

### Landmarks – The Importance of (1) Salience and (2) Other Modalities

Landmarks in our environment can be differentiated in regards to their (1) inherent features (*salience*) and the (2) sensory channel (*modality*) they trigger. In the following we want to differentiate between these two aspects and reason why we focus on modalities in wayfinding.

(1 – salience) The inherent features of landmarks can be described in terms of their salience, that is how much they stand out from the environment. Usually, a distinction between *structural* (e.g., location of the landmark), *visual* (e.g., contrast or size) and *semantic* salience (e.g., prominence or function) is made (Sorrows and Hirtle, 1999; Raubal and Winter, 2002; Klippel and Winter, 2005). Let us take the example of a statue serving as a landmark. If this statue is placed in front of an intersection in direction of a turn, it will in all likelihood have a high structural salience (Röser et al., 2012). If this statue is large and illuminated by spotlights in the dark, we could then speak of it as having a high visual salience. Finally, if this statue is famous and, more importantly, famous from that individual’s perspective (idiosyncratic), then we could speak of a high semantic salience. Especially in the case of semantic salience we can see that salience has to be more than just inherent features of an object. It strongly depends on the individual itself. Therefore, Caduff and Timpf (2008) not only emphasized the role of idiosyncratic relevance of a landmark but also, in contrast to the aforementioned distinctions of landmark salience, they differentiate between *perceptual* and *cognitive salience.*

However, despite their extensive efforts to demonstrate the inherent features of landmarks, a certain notion remained rather in the background. The authors claim that landmarks may be used in other modalities than just vision, but, unfortunately, only provide visual examples themselves:

“The most general requirement of a landmark is that it must be perceptually salient in some sense (i.e., visually, auditory, olfactory, or semantically). This requires […] a contrast with the environment (e.g., architectural differentiation), either in terms of its attributes (color, texture, size, shape, etc.) or due to its spatial location with respect to the other objects in the scene.” (Caduff and Timpf, 2008, p. 251).

(2 – modality) Thus, landmarks differ in the way they are perceived depending on which sensory channel they trigger. Logically, not all of our five sensory channels (visual, auditory, olfactory, haptic, and gustatory) are equally well-suited to process landmark information. Especially the gustatory channel is practically useless in regards to acting as a frame of reference in space. As initially demonstrated, research so far focused primarily on visual landmarks. Given the fact that we are predominantly optical beings it seemed to be an important and justified step.

Not only humans rely on landmarks as an important means for spatial orientation. Researchers have dealt with the question of how animals find their way for centuries (Darwin, 1873). Different topics were covered from the role of landmarks using bees (Tinbergen and Kruyt, 1938) and the assumption of cognitive maps (Tolman, 1948) to the importance of the hippocampus (O’Keefe and Nadel, 1978) in rodents. Using animals, more than just visual cues were examined in wayfinding processes. For example, in an experiment which was controlled for visual cues, it could be shown that echolocating bats can rely solely on acoustic landmarks (Jensen et al., 2005). There, echolocating bats found their way by using their biological sonar, i.e., auditory signals were reflected from an object and perceived acoustically. While the object itself did not emit the sound, the sensory channel that was used was auditory; hence, by definition, wayfinding was performed by means of an acoustic landmark. But, how would humans perform?

All these studies were conducted using animals. Their findings can be seen as an important contribution for the understanding of human wayfinding and as a basis of today’s research in landmark-based wayfinding. But, we cannot report such a massive transfer of knowledge of animal research when it comes to other modalities than vision. In humans, especially research in the field of ergonomics focuses on acoustic signals. For example, acoustic beacons were examined for their potential use in tunnels in case of an emergency (Boer and Withington, 2004). However, one novelty of our study lies in the fact that such beacons are not fully comparable to landmarks, which are embedded in another time frame. Beacons occur rarely and are therefore salient, whereas landmarks are permanently present. Therefore, they can be learned and are potential cues for wayfinding processes. Our study sets its focus on such landmarks which allows us to draw conclusions to wayfinding processes in our everyday lives. So far, we concentrated on single modality information processing. But, everyday life is much more complex than processing information in just one modality. We are throughout confronted with multi-sensory input, which is why we will demonstrate in the following section the importance of multimodal sensory information integration. Furthermore, the lack of literature examining multimodality in human landmark-based wayfinding needs to be addressed as well.

### Multimodality

Looking at one modality at a time allows us to understand the role of unimodal landmarks in wayfinding. But what about multimodality in wayfinding? In one experiment, Rossier et al. (2000) used the well-known Morris Water Maze (Morris, 1984). On the one hand, it could be shown that rats were not able to use up to three auditory cues in order to discriminate the goal location. On the other hand, the combination of two auditory cues and one visual cue resulted in sufficient spatial discrimination. Interestingly, place navigation was not supported any longer when the visual cue or the two auditory cues were removed.

We see that the integration of information from different modalities can indeed play an important role when it comes to spatial representations. The more important it seems to examine the role of different modalities in human wayfinding. In addition to the above presented literature, especially the following empirical findings built the foundation of our current study.

In our first experiment, we focused on unimodal processing. This topic was approached by Hamburger and Röser (2011, 2014) who addressed this issue by using a virtual environment. The authors compared wayfinding and recognition performances for acoustic and two types of visual (pictorial, e.g., an image of an object; verbal, e.g., the written name of the object) landmarks (unimodal presentation). In the recognition task, participants were tested whether they could recognize the landmarks they supposedly had seen or heard in the maze. On a descriptive level, performance was best for verbal landmarks; it was second best for acoustic landmarks and worst for pictorial landmarks. But the authors only found a significant difference between verbal and pictorial landmarks. In addition to that, when a modality switch was required (e.g., learning an image but being confronted with the according sound at test; incongruent modality), no so-called switching costs were found. This means that it did not take participants any longer to respond or resulted in any worse performance when the learned information (e.g., image of a frog) had to be translated into an acoustic signal at retrieval (e.g., croak of a frog).

In summary, performance was always worst when the initial landmark information had to be learned from pictures (independent of modality switch or not). More importantly, they found that wayfinding performance was not affected by the modality being used. However, it was important to see whether these findings can be partially replicated in our new experimental setting (e.g., virtual environment, head mounted display). The authors have used two types of visual landmarks: pictorial and verbal. We decided to use pictorial but not verbal landmarks in order to exclude language as a confounding component. Ideally, our findings allow comparisons to Experiment 2 in which we investigated the role of multimodality, congruency and learning strategies as part of our general discussion.

Another difference between the experiments conducted by Hamburger and Röser (2011, 2014) concerns the level of immersion. In their studies, they used computer displays and projection screens to present their three-dimensional virtual environment. Especially, if inter-individual differences are of great importance, then the degree as to which physics are represented correctly in a virtual environment is crucial. For this we used a state of the art technique and allowed participants to freely move their upper body part and head within the virtual environment, realistic sound physics as well as shadow and lighting conditions.

In our second experiment, we focused on multimodal processing. This topic was addressed by Werkhoven et al. (2014). The authors presented either visual, acoustic or a combination of audio and visual landmarks. Participants in the audio-visual landmark condition were not only better in recalling landmarks but also in wayfinding. Our experiment tried to build on their work in the following ways. First, we also wanted to investigate the role of multimodal landmark information and also planned to present acoustic and visual landmarks simultaneously. Second, Werkhoven et al. (2014) were partially motivated by the role of meaning in multisensory landmarks. If meaning is that important, the semantic congruency might be important as well. Also, with respect to our study, we needed to address some methodological aspects. For example, while their study allowed free movement of the avatar in space by using a keyboard, it contained other limitations. Participants had 90 s to explore the full maze, which does not allow to control for landmark exposure times. Also, in the wayfinding task, participants were placed somewhere in the maze and had to find their way to a given landmark. Such paradigms test for survey knowledge. On the downside, they do not allow to control for different encoding strategies, such as route encoders, who report to learn direction sequences. Same as it was the case for the studies conducted by Hamburger and Röser (2011, 2014) the authors used regular LCD displays. Finally, the presentation of auditory information was connected to visual presence of a landmark. The sound was presented for 3.5 s when the landmark was within the field of view. And it was played again after an interval of 0.5 s if the landmark was still within the field of view. However, we assume that, for example, dogs do not suddenly stop barking when we turn our head around.

### General Assumptions and Aim

As we have seen, spatial representations are a result of multisensory information integration (Kelly and Avraamides, 2011). Stein et al. (1996) already showed that the intensity of visual stimuli was influenced by the presence of non-visual (auditory) stimuli. More recent findings suggest that the multisensory information processing of a scene can be facilitated when paired with a semantically congruent auditory signal (Tan and Yeh, 2015). This congruency effect has been taken as evidence that audio-visual integration occurs for complex scenes. As navigation in our environment consists of a seamless integration of complex sceneries, a fundamental question arises: how is human wayfinding affected by multimodality?

All these mixed findings lead us to the hypotheses that (1) there is no superior modality *per se* and that (2) the role of multimodality in landmark-based wayfinding might depend on object-related (e.g., congruency) and subject-related factors (e.g., cognitive style). Since there are only a few studies available on different sensory modalities in regards to human landmark-based wayfinding, we here investigate these assumptions in a series of two experiments in order to provide much better insights into modality-specific and multimodal processing of landmark information.

## General Methods

In this section we will describe methodological aspects which both of our experiments have in common. Both were conducted in accordance with the declaration of Helsinki. More specific experiment-related aspects will be described in each experiment-section individually.

### Participants

Participants in the two experiments were in sum 51 students from the Justus Liebig University of Giessen ranging from 19 to 35 years (*M* = 24.10, *SD* = 3.52). All participants provided informed written consent and received either course credits or money for compensation. Participants who reported colorblindness, epilepsy or epilepsy in close relatives were excluded from the study. Since we did not investigate any gender differences and exploratory analyses from earlier studies did not result in significant gender differences, gender will not be reported from hereinafter.

### Materials

**FIGURE 1:**
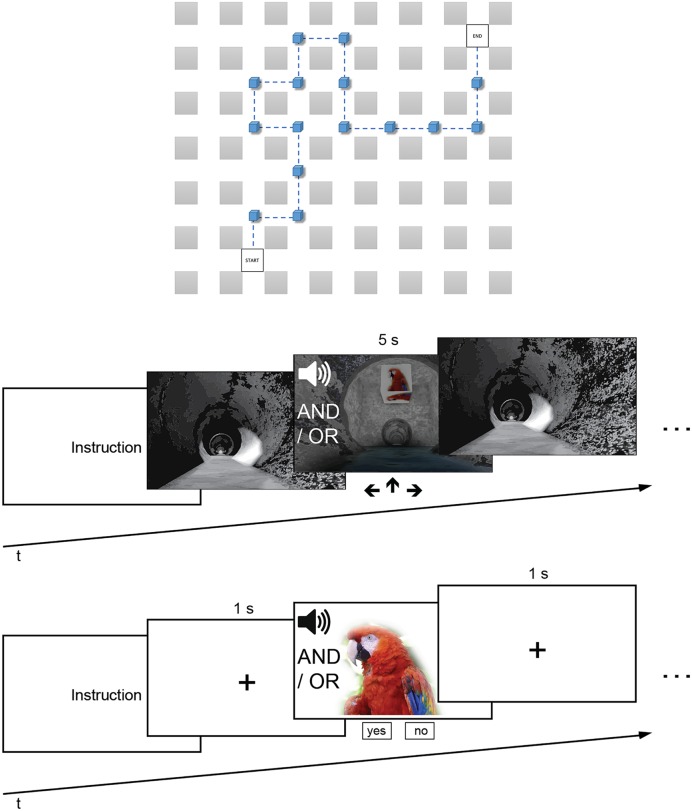
**(Top)** Exemplary route of the wayfinding task from a bird’s-eye view. **(Middle)** Wayfinding task with acoustic and/or visual landmarks. **(Bottom)** Recognition task in which participants indicated whether they had seen and/or heard the landmark or not.

The wayfinding task was conducted in a virtual environment with a head-mounted-display (HMD). The virtual environment consisted of a sewer system with a simple grid structure (see **Figure 1**). The intersections had a length of 375 cm and were connected by tunnel segments each with a length of 650 cm. In order to control for directional biases three routes were programmed so that each landmark is associated once with each direction (left, straight, right). The virtual environment was programmed with Unreal^®^ Engine 4, which was run on a Pentium i7 3.6 GHz computer (16 GB RAM, running Windows 8.1). It was displayed on an OLED HMD that also allowed positional head tracking (Oculus Rift^TM^ Developmental Kit 2, Oculus VR, LCC). To each eye, a resolution of 960 × 1080 pixels was technically visible (Full HD).

Our experiment is in line with well-established wayfinding paradigms (e.g., Strickrodt et al., 2015). It could be assumed that participants in these types of virtual-reality wayfinding experiments do not perform any wayfinding but rather some type of sequential learning or list learning, i.e., encode only direction sequences such as right, right, left, straight, right, and so on. However, this has been ruled out in several previous experiments (e.g., Balaban et al., 2014; Hamburger and Röser, 2014) where participants were controlled for sequential learning with random trials (e.g., virtually beamed into random positions in the maze and answered questions about this intersection), demonstrating that they at least encoded a path consisting of intersections with landmarks *and* directions. *Pure* sequential learning could also not explain the high landmark recognition performance obtained in these experiments.

Fifteen animal pictures and corresponding sounds served as landmarks. It should be mentioned that landmarks, by definition, can only serve as reference points because they do not change their position. For example, a dog would be a poor landmark in the real world. But, because we present static images, animal pictures could serve as landmarks. All sounds and pictures were part of Creative Commons (CC). These type of public copyright licenses are used when authors permit the use of their work under certain conditions (e.g., non-commercial use). The pictures were placed in the middle of an intersection at a height of 220 cm. In conditions in which acoustic landmarks were available, the invisible sound emitter was placed at the same position. In order to give a more realistic feeling of a sound emitter (e.g., dog) a dynamic sound model was applied allowing a logarithmic reduction in volume over distance. That is to say, the lowest volume was in the middle of a tunnel segment, it increased while subjects approached an acoustic landmark until the center of an intersection and decreased again until the middle of the next segment. We accepted the fact that the presentation times of acoustic landmarks are therefore longer than those for visual landmarks in exchange for an increase in ecological validity (sound emitter are not suddenly muted when we turn our back on them).

Participants wore noise-canceling headphones (Bose^®^ Quiet Comfort^®^ 25 Acoustic Noise Cancelling^®^) throughout the entire wayfinding task.

The recognition task was conducted in MATLAB^®^ Release 2014b (The MathWorks, Inc., Natick, MA, USA) in connection with the Psychophysics Toolbox (Brainard, 1997; Pelli, 1997; Kleiner et al., 2007). Participants were positioned in front of a 21.5 inch liquid crystal display monitor (Dell, Inc., Round Rock, TX, USA) with a distance of 60 cm. The stimuli used were the same as in the wayfinding task. Additionally, the same number of distractors were added.

### Procedure

Summarized, both of our experiments (Experiment 1: unimodal; Experiment 2: multimodal) consisted of a wayfinding and recognition task. The wayfinding task had two phases. In the first phase (learning) participants passed the landmarks once and had to learn the route. In the second phase (wayfinding) participants were stopped at each intersection (right before a landmark) and had to indicate the correct direction. Subsequently to the wayfinding task the recognition task was conducted in which participants had to decide whether a given landmark was present along the route or not. In the following section we will explain the procedure in detail.

Before the experiments began, it was again ensured that participants did not meet the exclusion criteria. Thereafter, the HMD was calibrated and each participant was again tested for visual and auditory acuity with the HMD on. Both experiments consisted of three phases, namely encoding, wayfinding, and recognition (**Figure 1**).

At the beginning of the experiment, instructions were presented to participants visually and acoustically. In this text (presented in German) they were told that (1) they will be guided through a tunnel system in a first run and that (2) there will be a second run in which they will stop at each intersection and will be asked for the correct route. During the encoding phase, participants had to learn the route while they were auto piloted through the maze. This phase was finished shortly after passing all 15 landmarks.

The wayfinding phase also began with an audio-visual instruction of the task. In this instruction text (also presented in German) they were told that the second run (along the same route) was going to start and that they would have to indicate the correct direction at each stop by pressing a corresponding button. They were also told that, regardless of their input, the correct route would be continued after 5 s. Participants were then again auto piloted through the maze. This time, however, the auto pilot stopped directly in front of each intersection and participants had to indicate within 5 s the correct direction (left, right, or straight) by pressing the corresponding arrow key. One reason for limiting the exposure time was to avoid confounding effects in the subsequent phase two: the recognition task. Another reason was that it might have prevented an unlimited rehearsal and list learning. Regardless of their input, the correct route was continued until again all 15 landmarks were passed (implicit feedback). The reason for using this kind of autopilot and route continuation was to prevent participants from getting lost. This is a passive form of testing wayfinding performance and allowed comparability to earlier findings (Hamburger and Röser, 2014) and control stimulus exposure times.

The recognition phase started with an instruction text and participants could indicate by pressing the space bar when they were ready to continue. Each trial began with a fixation cross that was presented for 1000 ms. Then a landmark was presented and participants could indicate whether they had seen or heard that particular landmark by pressing keys for yes and no respectively. The next trial began directly after a decision was made or when no decision was made after 5000 ms.

In sum, each participant was tested for 30 landmarks (15 target stimuli and 15 distractors) which were randomly presented in the recognition phase. Afterward, participants were asked to fill out a questionnaire in which (besides basic demographics) hiking experience, experience with navigation systems and virtual environments (gaming) had to be indicated. We assumed that these factors could be relevant for navigation and potentially influence our results. For example, one of our preliminary studies showed that the amount of hours participants play action-adventure games or strategy games correlates negatively with wayfinding performance. This could not be found for genres in which a first-person perspective is used (e.g., first-person shooters).

In order to include cognitive styles into our analysis, we decided to assess the encoding strategies of our participants afterward. Participants had to answer the question “How did you memorize the correct route?” as part of the questionnaire. This was followed by an interview in which we elaborated on our participants’ reports to ensure the correct categorization into encoding strategies. We differentiated between object encoding strategies (memorizing the landmarks) and route encoding strategies (memorizing sequences of directions). The experimenter was instructed to let the participants describe how they memorized the route in their own words. Then participants were asked to describe specific strategies of object learners (focusing on landmarks) and route learners (memorizing direction sequences only) and whether they saw themselves using one strategy only. The experiment was conducted by one experimenter who also had to take notes of all responses during the interview. In total we had three elements to categorize a participant: (1) the answer of the questionnaire, (2) experimenter’s notes, and (3) experimenter’s presence during categorization. If participants were indifferent or described mixed strategies, we did not assign them to one of those two groups.

## Experiment 1 – Unimodal

### Overview and Methods

Our aim in Experiment 1 was to show that there is indeed no superior modality when it comes to landmark-based wayfinding. We expected null-effects for both wayfinding and landmark-recognition performance. Our general methods were described above. In this experiment, specifically, 28 participants were randomly assigned to one of two groups of the factor landmark type (two levels: *acoustic landmarks* and *visual landmarks;* between-subject design). The data of five participants were excluded from further analysis, since they did not exceed chance level in either the wayfinding or the recognition task. This resulted in 23 participants (mean age = 24.09, ranging from 19 to 35 years) from which 11 participants were in the acoustic landmark condition (ALC) and 12 in the visual landmark condition (VLC).

In the ALC, sound emitters were placed on each intersection but no visual landmarks were presented. The other group (VLC) had to perform the wayfinding task by using visual landmarks but did not receive any auditory landmark information.

During the recognition task, participants of the VLC had to indicate whether they had seen the presented picture during the encoding and wayfinding stages or not. The recognition task in the ALC did not differ from the VLC group, except that, instead of pictures, sounds were presented while the screen remained black.

### Results

**FIGURE 2:**
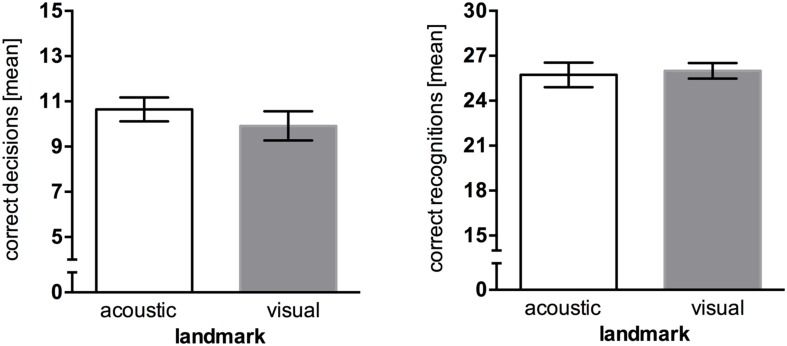
**Results of Experiment 1, error bars represent the *SEM*. (Left)** Mean correct route decisions of the wayfinding task (chance level = 5). **(Right)** Mean correct answers of the recognition task (chance level = 15).

The results show that wayfinding performance for acoustic landmarks (*M* = 10.64, *SEM* = 0.53) was only marginally better than for visual landmarks (*M* = 9.91, *SEM* = 0.65). An independent two-tailed *t*-test revealed no significant difference in wayfinding performance between different landmark types, *t*(21) = 0.855, *p* = 0.402. The landmark type did not affect recognition performance as well, *t*(21) = -0.285, *p* = 0.778, with acoustic landmarks (*M* = 25.72, *SEM* = 0.82) having an almost equal recognition rate compared to visual landmarks (*M* = 26.00, *SEM* = 0.52; **Figure 2**). A one-sample *t*-test against 30 was conducted to compare the recognition task results to performance ceiling. The results show a significant difference, *t*(22) = -8.834, *p* < 0.001.

### Discussion

In the unimodal landmark condition, acoustic landmarks served as well as visual landmarks for wayfinding and landmark recognition in the virtual tunnel system. As expected, this finding is in line with the results of Hamburger and Röser (2011, 2014). Moreover, the idea that auditory cues could serve equally well could also be shown for other tasks. The findings of Werkhoven et al. (2014) strengthen the notion of modality equality by demonstrating equal performances when participants have to (1) recall landmarks (free recall), (2) recall adjacent landmarks when one landmark was given (cued recall) or (3) draw memorized route segments. If using such paradigms in a virtual environment allows us to draw conclusions to real life (see General Discussion), then these findings could also demonstrate that acoustic landmarks are valuable not only for blind people during wayfinding but also for normally sighted people.

Equal results for both modalities could also be interpreted in another way. Participants could just have ignored the landmarks and learned sequences of directions. We do not believe that this is the case. Especially from performances in the recognition task we draw the conclusion that participants have learned the landmarks. If this were not the case and they did not process the landmarks (i.e., missed or successfully ignored them during encoding), then recognition performance would not have differed significantly from chance level.

Since we now have an idea of how well these (visual or acoustic) objects can serve as landmarks in a spatial context, we now need to investigate what happens if spatially relevant information is available in two modalities, namely acoustic and visual (same = congruent; different = incongruent).

## Experiment 2 – Multimodal

### Overview and Methods

Experiment 2 now investigated the role of multimodal information processing in landmark-based wayfinding. It was based on Experiment 1, but this time the wayfinding and recognition task consisted of audio-visual landmarks. In addition to Experiment 1, the recognition task was now designed to assess response times as well and we furthermore explored encoding strategies by interviewing each participant afterward. In the recognition task, the landmarks were presented in the same combinations as they were presented in the maze.

From 23 new participants, two had to be excluded due to not exceeding chance level in one of the two tasks, either wayfinding (5 correct decisions = 33%) or recognition (15 correct decisions = 50%). Twenty-one participants (mean age = 24.33, ranging from 20 to 33 years) were randomly assigned to either the congruent (*n* = 10) or incongruent (*n* = 11) condition. The first consisted of landmarks which were combinations of pictures with a semantically congruent sound (e.g., barking dog) while in the latter case, the sounds did not match the picture (e.g., a picture of a dog presented with the sound of a pig). The same animal did not appear in different combinations in order to avoid confusion.

### Results

Again, the means of correct decisions were calculated and compared with a two-tailed *t*-test. Mann–Whitney *U*-test was used as a non-parametrical equivalent when needed. Any correlations reported were conducted by using Spearman’s Rank Order Correlation.

**FIGURE 3:**
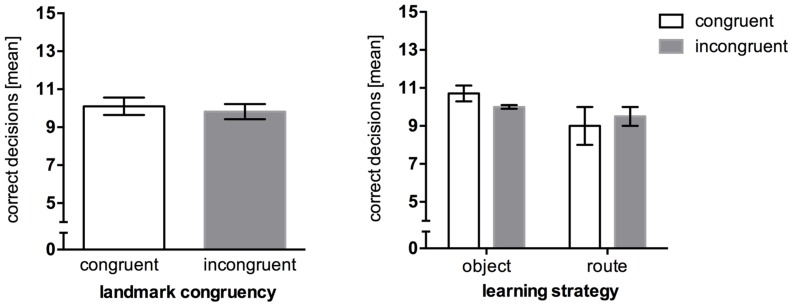
**Mean correct route decisions in the wayfinding task of Experiment 2, error bars represent the *SEM*, chance level = 5. (Left)** Comparison between wayfinding performance in the congruent landmark condition and the incongruent landmark condition. **(Right)** Wayfinding performance between congruency levels after *post hoc* categorization of strategies.

Wayfinding performance in the congruent condition (*M* = 10.10, *SEM* = 0.45) was almost on the same level with the incongruent condition (*M* = 9.82, *SEM* = 0.40), *U* = 3.00, *p* = 0.800. Taking the reported strategies (congruent: seven object encoders, two route encoders, one no single strategy reported; incongruent: two object encoders, four route encoders, five no single strategy reported) into account, one could precautiously assume that object encoding strategies could be superior to route encoding strategies (**Figure 3**). However, no significant differences were obtained in regards to wayfinding as well.

**FIGURE 4:**
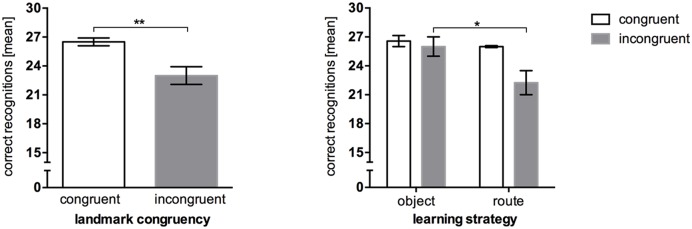
**Mean correct recognition performance in Experiment 2, error bars represent the *SEM*, chance level = 15. (Left)** Comparison between recognition performance in the congruent landmark condition and the incongruent landmark condition. **(Right)** Recognition performance between congruency levels after *post hoc* categorization of strategies. ^∗^*p* < 0.05; ^∗∗^*p* < 0.01.

A one-sample *t*-test against 30 was conducted to compare the recognition task results to performance ceiling. The results show a significant difference, *t*(20) = -8.367, *p* < 0.001. Performance in the recognition task differed significantly in regards to congruency level, *U* = 17.00, *p* = 0.006. Congruent landmarks were recognized better (*M* = 26.50, *SEM* = 0.40) than incongruent landmarks (*M* = 23.00, *SEM* = 0.92; **Figure 4**). However, object- and route encoders showed significantly different recognition performance only when incongruent landmark combinations were used, *U* = 9.00, *p* = 0.036.

**FIGURE 5:**
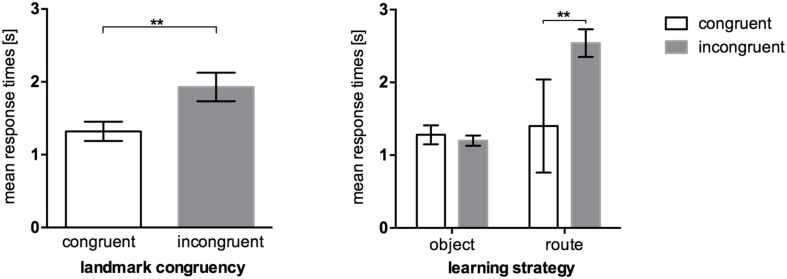
**Mean response times of the recognition task in Experiment 2, error bars represent the *SEM*. (Left)** Comparison between response times in the congruent landmark condition and the incongruent landmark condition. **(Right)** Response times between congruency levels after *post hoc* categorization of strategies. ^∗∗^*p* < 0.01.

The same pattern could be shown for response times, *t*(17.138) = -2.577, *p* = 0.019. Participants were faster in recognizing congruent landmarks (*M* = 1.32 s, *SEM* = 0.13) than incongruent landmarks (*M* = 1.93 s, *SEM* = 0.20; **Figure 5**, left). Again, taking the reported strategies *post hoc* into account (**Figure 5**, right), we see that object encoders were almost equally fast regardless of the congruency of an audio-visual landmark. Congruency affected only route encoders. In order to test this interaction hypothesis we conducted a 2 × 2 analysis of variance which resulted in a significant congruence*strategy interaction, *F*(1,11) = 5.951, *p* = 0.033, $\eta_{p}^{2}$ = 0.351. Pairwise comparisons reveal that congruent landmarks were recognized faster (*M* = 1.40 s, *SEM* = 0.64) than incongruent landmarks (*M* = 2.54 s, *SEM* = 0.19) in route encoders, *p* = 0.004 (Bonferroni corrected).

Analysis of the exploratory questionnaire did not reveal any significant effects, except that participants who reported to go hiking on a regular basis performed significantly better in the wayfinding task compared to those who reported to do not so, *U* = 13.00, *p* = 0.025.

### Discussion

On the one hand, in wayfinding, congruent and incongruent landmark information led to the same performances. Even if we include the reported learning strategy in our analysis there are no effects. This finding suggests that the superiority of audio-visual landmark presentation demonstrated by Werkhoven et al. (2014) does not lie in its congruency but more on the fact that both sensory channels are triggered.

On the other hand, in the recognition task, participants of the congruent condition outperformed those of the incongruent condition. This shows that multisensory information processing can indeed be facilitated when two semantically congruent signals are combined (Tan and Yeh, 2015). Additionally, incongruent landmark information required the longest time being processed. This was also found in other areas of psychological research. For example Laurienti et al. (2004) used a cue feature discrimination task. In this task, participants were confronted with visual (e.g., red or blue circle), auditory (e.g., verbalizations of the word red or blue) or both stimuli simultaneously. They were told, for example, to respond to one color with the index finger and to the other color with the middle finger. Similar to findings in our study, the authors found elongated response times when semantically incongruent stimuli were combined in a multimodal setting. Besides the differences between the experimental paradigms of our studies we have to pay due attention to the importance of semantic congruence.

But, we believe that the results as stated above do not give credit to what the data really contain. In Experiment 2 we introduced the concept of cognitive style (e.g., Kato and Takeuchi, 2003; Pazzaglia and Moè, 2013; Wedell et al., 2014) in that we had participants report their encoding strategy: either memorizing the landmarks (object encoding) or memorizing sequences of directions (route encoding). In wayfinding, object encoders performed slightly better independent of the congruency of the information on a descriptive level, however, these differences were not significant.

For the recognition phase we see strong effects of encoding strategies: the lack of congruency of a visual-acoustic landmark combination only affected route encoders. One could now argue that this result is not surprising at all, since encoding the route only could imply that they simply ignored the objects. But, what then about the route encoders in the congruent condition? For them this argument counts as well, but performance was obviously not reduced in any way.

The findings of the recognition task are also supported by the response times. As expected, congruent landmark information is processed faster compared to incongruent landmark information. The reason for the elongated response times in the incongruent group is due to the route encoders, who required almost double the amount of time to give a response in comparison to the route encoders in the congruent group. Thus, correct recognition and response time complement each other perfectly well. So, multimodal integration takes place even without conscious awareness. In other words, even if we believe to apply a certain strategy (i.e., memorizing route directions only), we are still able to recognize the required information (i.e., landmarks).

## General Discussion

In a series of two experiments, we examined the role of multi-sensory information integration in human landmark-based wayfinding. The first experiment was focused on unimodal landmark information. We therefore compared wayfinding and recognition performance in a virtual environment by using either acoustic or visual landmarks. The results supported the assumption that there is no superiority of one modality in regards to landmark-based wayfinding as suggested in terms of visual superiority in past research (e.g., Presson and Montello, 1988).

The second experiment was focused on multimodal landmark information integration. Again, wayfinding and recognition tasks were performed. In addition to that, response times and encoding strategies were measured. We could show that congruent audio-visual landmark information can be processed more effectively as they were recognized better and faster (congruency effect; Tan and Yeh, 2015) than incongruent landmarks. Looking deeper into the reported strategies, we could now say that the missing congruency of an audio-visual landmark affects primarily those who learn sequences of directions (route encoders). This would support our assumption that the role of multimodality in landmark-based wayfinding depends not only on object-related but also on subject-related factors.

### Semantic Congruence and Multimodal Landmarks

The object-related factors presented in this study seem to be crucial for multimodality in human landmark-based wayfinding. The question is: why is congruency of such importance? We know that *novelty* can be an important factor when it comes to encoding new information (for a review see Nyberg, 2005). Therefore, we could also assume that if we do not expect a dog to grunt, then we would memorize a grunting dog (which would be a novel and therefore highly salient event) more efficiently. In contrast, our results demonstrate that incongruent landmarks did either not affect recognition performance (for object encoders) or worsen it negatively (for route encoders). The results can be seen as evidence for an important role of semantic networks and multimodal integration in landmark recognition. This could explain why those who believed to have not payed attention to audio-visual landmarks (route encoders) were still able to recognize congruent landmarks compared to incongruent landmarks.

Another limitation of our study is that we could not control landmark salience in all aspects. While visual landmarks can be controlled for several types of landmark salience, it cannot be fully controlled for semantic salience, especially because of the idiosyncratic relevance. For example, a dolphin might be associated with the feeling of happiness due to memories of the last vacation. The salience of acoustic landmarks is not well-examined yet. From research in the field of ergonomics we know that the quality judgment of an acoustic signal is highly negatively correlated to localization errors (Tran et al., 2000). The authors could show, for example, that non-speech beacons were preferred over speech beacons. Also the problem of semantic salience remains for acoustic landmarks.

While in this study we focused on two modalities (auditory and vision), it should be mentioned that other modalities can indeed be used in order to help wayfinding. For example, Rossier and Schenk (2003) could show that olfaction enables the use of visual cues in 48-day-old rats. Another study found evidence for the role of magnetic cues in wayfinding by attaching magnets to the head of king penguin chicks (Nesterova et al., 2013). In the long run, it is important to see whether human wayfinding can also be improved by using olfactory cues.

### Self-Reported Strategies

It must be pointed out that the findings for subject-related factors are associated with some limitations. The object-related factors were not only defined *a priori* but were also considered in our experimental paradigm. We combined semantically congruent and incongruent audio-visual stimuli and defined congruency as our between-subject factor. The subject-related factors (strategies) were *post hoc* measures and can be seen as a novelty in combination to our object-related factors. It was, therefore, particularly important to apply strong criteria for categorizing participants into object- and route-encoders. We did so only if we were certain that a participant reported a single strategy. On the downside, applying such strong criteria led to a relatively small number of participants who actually reported a single strategy only. People who explicitly reported both strategies or were indifferent about it were not part of the analysis. Certainly, they did use some kind of strategy but probably did not do so on purpose. Could this imply that those who choose a certain strategy beforehand encode differently than those who do not? To answer this question, considering its complexity, more research in the field of spatial cognition and individual differences has to be conducted. We therefore understand our findings in regards to the role of self-reported encoding strategies as an important indicator pointing in a certain direction. Future studies, could use preliminary experiments in order to (1) see whether self-reported strategies are robust (do participants report different strategies per trial?) and (2) if that is the case, categorize them accordingly in order to use this information as a between subject factor *a priori*. Finally, the term self-reported strategy implies that the encoding strategies here are subjective. Another interesting aspect could be to validate subjective measures with objective measures such as eye-tracking (e.g., de Condappa and Wiener, 2016). This could lead to a better understanding of how such beliefs are made and how they influence behavioral performance.

### Virtual Reality and the Real World

Technological progress and ubiquitous presence of virtual environments in different areas (video games, surgery, etc.; Karimpur and Hamburger, 2015) raise the question of ecological validity when using such an environment in wayfinding experiments. In research, we always have to find a perfect balance between an acceptable level of experimental control and realism. In this study the use of a virtual environment was crucial for conducting the experiment (semantically incongruent multimodal stimulus presentation, etc.). Studies demonstrate that even with technology which is today seen as antiquated, participants were able to develop route-finding abilities and survey-knowledge almost as accurate as people who developed these in a real environment (Ruddle et al., 1997). More importantly, Lloyd et al. (2009) could show that the assumed equivalence between virtual environments and the real world does not only hold for route-learning but also for strategy preferences such as using landmarks, creating a bird’s-eye map, etc.

Wayfinding is more complex than one might think, however, these findings support the methodological approach used here. Yet, one aspect limits our findings as well. In order to compare our findings with those of Hamburger and Röser (2014) we decided to use an autopilot and correct route continuation. This prevented participants from getting lost. It additionally allowed us to control exposure times. In consequence, participants were restricted in the strategies they could have used. For example, they were not able to walk freely around and look down each path in order to use a *view-matching* strategy. While for other research questions, the ability to use every possible strategy can be important, for our experiment it was not. We believe that both types of paradigms have their advantages and disadvantages. It is mainly the research question that determines which paradigm to chose. Our results emphasize all the more the need to consider different methodological approaches in order to investigate the role of modalities, how information is combined and the importance of cognitive styles in human landmark-based wayfinding.

## Author Contributions

HK has been involved in designing the study, developing the virtual environment, data collection, data analysis, and writing the manuscript; KH has been involved in designing the study and writing the manuscript

## Conflict of Interest Statement

The authors declare that the research was conducted in the absence of any commercial or financial relationships that could be construed as a potential conflict of interest.
